# Late Gestation Maternal Feed Restriction Decreases Microbial Diversity of the Placenta While Mineral Supplementation Improves Richness of the Fetal Gut Microbiome in Cattle

**DOI:** 10.3390/ani11082219

**Published:** 2021-07-27

**Authors:** Gwendolynn Hummel, Kelly Woodruff, Kathleen Austin, Ryan Knuth, Scott Lake, Hannah Cunningham-Hollinger

**Affiliations:** Department of Animal Science, University of Wyoming, Laramie, WY 82071, USA; ghummel@uwyo.edu (G.H.); klomagno@uwyo.edu (K.W.); kathyaus@uwyo.edu (K.A.); rknuth@uwyo.edu (R.K.); scott.lake@uwyo.edu (S.L.)

**Keywords:** microbiome colonization, next generation sequencing, reproductive and fetal microbiome, undernutrition

## Abstract

**Simple Summary:**

The purpose of this study was to explore the influence of maternal feed intake restriction on the microbiome of the reproductive tract and its subsequent colonization of the bovine fetal gut microbiome in utero. After sequencing the microbial DNA of the maternal vagina, placental tissues, and fetal gut, our data show the microbiome of the vagina and cotyledon remain relatively unaffected by feed intake restriction and mineral supplementation. The placental microbiome of feed restricted pregnancies was less diverse, and feeding using a mineral supplement did not impact these differences. However, mineral supplementation improved the richness of the fetal gut microbiomes regardless of feeding treatment, substantiating prior evidence that in utero mineral supplementation improves calf performance. The obtained results may offer insight into improving nutrient use of calves born to dams experiencing feed restriction.

**Abstract:**

Feed intake restriction impacts both humans and ruminants in late gestation, although it is unknown whether this adverse maternal environment influences the microbiome of the reproductive tract, and through it, the colonization of the fetal gut. A 2 × 2 factorial design including a 70% feed intake restriction (feed restricted ‘FR’ or control diets ‘CON’) and mineral supplementation (unsupplemented ‘S−’ or supplemented ‘S+’) was used to analyze these effects in multiparous cows (*n* = 27). Vaginal swabs were obtained 60, 30, and 10 days prior to the estimated calving date, along with neonatal rumen fluid and meconium. Placental tissues and efficiency measurements were collected. Microbial DNA was extracted for 16S sequencing of the V4 region. Feed restriction decreased the diversity of the placental microbiome, but not the vagina, while mineral supplementation had little impact on these microbial communities. Mineral supplementation did improve the richness and diversity of the fetal gut microbiomes in relation to reproductive microbes. These differences within the placental microbiome may influence individual health and performance. Adequate maternal nutrition and supplementation yielded the greatest placental efficiency, which may aid in the establishment of a healthy placental microbiome and fetal microbial colonization.

## 1. Introduction

While it is known that intrauterine exposure to undernutrition elicits long-term consequences for offspring [1,2], studies evaluating the effects of this maternal environment upon fetal microbiome colonization have received little attention. This is despite the fact that feed intake restriction (FR) during gestation is a growing concern for human health, where the prevalence of suboptimal body mass index during pregnancy has increased in both the US [3] and China [4]. Ruminants also experience voluntary FR during late gestation, as both fetal growth and abdominal fat impede upon rumen volume and compete for space in the abdomen [5,6]. As the volume of these incompressible abdominal contents increases, rumen volume decreases along with voluntary feed intake. Ruminal capacity has been reported to only be reduced by 5% 61 days prepartum, but dry matter intake may be depressed by as much as 69% [7]. This may be because dry matter and water intake are most affected by gut fill restrictions [8], although rumen liquid volume and kinetics are not altered. Reduced feed intake may be aggravated during the winter months, which primarily coincide with late gestation in the Mountain West region, when nutrient requirements are at their highest and foraging availability and quality are limited [9]. These perturbations to maternal feed intake in late gestation may result in decreased growth rates and suboptimal carcasses in offspring [10] and may diminish offspring growth even in the absence of birth weight differences [11,12].

Maternal undernutrition often impedes fetal and postnatal growth in humans [13] and cattle [14], where ruminants experience impaired skeletal muscle development and a predisposition to fat accumulation [15], ultimately impacting meat quality [16]. It is unknown whether the development of the fetal gut microbiome, whose inoculation has been widely demonstrated to begin in utero [17,18,19,20,21,22,23], is similarly impacted by maternal diet. If so, any programming effects of this early microbiome may carry similarly severe consequences for lifelong growth and metabolism.

It has been demonstrated that FR does alter the gut microbiome of mature ruminants. The rumen of FR beef steers experienced an increased relative abundance of archaeal spp. and a reduction of *Succinivibrio*, a genus that is typically responsible for reducing methane emissions and improving feed efficiency [24]. Even neonatal lambs exposed to FR postpartum displayed an increase in *Prevotella* spp. in the ileum, which may promote inflammation and dysbiosis [25]. The rumen of FR ewes in late gestation have also demonstrated an increase in several bacterial populations that typically occupy a low relative abundance in this microbial community [26], potentially altering the microbial environment of the mature rumen to effect host health and performance.

The human gut microbiome is also altered by FR in adulthood [27,28], and differences between appropriate and excessive gestational weight gain illustrate that the placental microbiome may reflect the maternal gut microbiome [29], offering an avenue by which maternal diet may influence fetal gut inoculation [23,30]. While excessive gestational weight gain has been shown to cause aberrations to the placental microbiome in humans, the effects of insufficient gestational weight gain remain to be explored.

These findings within the human microbiome may be extended to other species. In both ruminants and humans, a strong correlation exists between fetal weight and placental size when maternal nutrient consumption is challenged [1,31]. A similar correlation may exist between placental efficiency and microbiome colonization, as microbes may gain access to the placenta through hematogenous transfer [32,33] via the cotyledon. In rats, the placenta has been suggested to increase efficiency during maternal protein restriction in order to maximize substrate transfer to the fetus and maintain normal fetal growth in late gestation [34]. Increasing cotyledonary surface area, and therefore placental efficiency, may not only maintain fetal growth in cattle, but may alter colonization patterns of the fetal gut microbiome via the placenta in late gestation.

Therefore, the present study sought to characterize the efficiency and microbial composition of the bovine placenta after a period of feed intake restriction (FR) compared to the placentae of control (CON) cows. Because the vaginal microbiome has been suggested to contribute to placental microbial load [35] and even to the fetal gut [36,37], we sought to characterize the vaginal microbiome throughout the last third of gestation to evaluate its role in contributing to the fetal gut microbiome as well as to determine any effects of nutrition on these microbial communities. Finally, we asked whether the placental or vaginal microbiomes are subject to community changes due to mineral supplementation and if these changes are compounded by feed intake restriction.

## 2. Materials and Methods

### 2.1. Animals and Study Design

Three months prior to their expected calving date, university multiparous Angus cross-bred cows (*n* = 60) were stratified by age, weight, and body condition score (BCS) into a 2 × 2 factorial design. Maternal BCS was collected at the start of the study and every subsequent two weeks until 1 month postpartum. Cow body weight was obtained 60 ± 10, 30 ± 10, and 10 ± 10 days prior to their expected calving date. These samples were taken an average of 64, 34, and 14 days from their respective calving date.

The study design included a 70% intake restricted diet (feed restricted ‘FR’ or control diets ‘CON’), which not only provides a sufficient dietary restriction, but also reflects a level of restriction most commonly experienced by beef herds in the western USA during late gestation [38]. A mineral supplement [39] (Availa 4, Zinpro Corporation, Eden Prairie, MN) was included (unsupplemented ‘S−‘ or supplemented ‘S+’) one month prior to the expected calving date. Cows were provided ad libitum access of up to 0.91 kg×hd^−1^×day^−1^.

This resulted in four treatment combinations: feed restricted with mineral supplementation (FR/S+; *n* = 15), feed restricted without mineral supplementation (FR/S−; *n* = 15), control diet with mineral supplementation (CON/S+; *n* = 15), and control diet without mineral supplementation (CON/S−; *n* = 15). Sub-samples of = 8 per treatment group were used for microbial analysis.

### 2.2. Vaginal Microbiome Sample Collection

Vaginal swabs were obtained 60 ± 10 days (Vag60; *n* = 32), 30 ± 10 days (Vag30; *n* = 32), and 10 ± 10 days (Vag10; *n* = 30) prior to the expected calving date. The vulva and perineal area were disinfected, and swabs of vaginal epithelium were obtained using sterile double-sheathed equine uterine culture swabs [40] (Jorgenson Labs, Loveland, CO, USA). Each swab was inserted to the midpoint of the vagina, exposed from the sterile sheath, and rotated for maximum contact before retraction into the sheath.

### 2.3. Fetal Gut Microbiome Sample Collection

Following parturition, rumen fluid and meconium were collected from each calf (*n* = 27) before the neonate was permitted to suckle. Rumen fluid was collected by passing a lubricated stomach tube orally to the rumen and applying suction via syringe so that 20–30 mL of fluid was aspirated [41]. A sterile double-sheathed equine uterine culture swab was inserted rectally in order to collect meconium immediately following parturition [20]. Each sterile swab was inserted roughly 2.5 cm into the rectum of the newborn calf, and after the cotton tip was exposed, rotated before retraction into the sheath and removal from the rectum.

### 2.4. Placental Efficiency Measurements

Immediately following expulsion, whole placentae (*n* = 27) were collected and inspected for completeness. The entire chorioallantois was weighed and oriented so that the gross macroscopic characteristics could be analyzed, and the placenta was determined to be intact. Each cotyledon was laid flat along the intercotyledonary membrane (ICM), and the major and minor diameters were measured using calipers with an accuracy of 1 mm. Following the protocol of Van Eetvelde et al. [42], individual cotyledonary surface area was calculated as

(1) Area (ellipse)=πab

where *a* = half the major diameter, and *b* = half the minor diameter. Total cotyledonary surface area was calculated as the sum of each individual cotyledon’s surface area per placenta. Accessory cotyledons [42] (<10 mm in major diameter) were excluded from cotyledonary surface area calculations. Placental efficiency was therefore determined as birth weight/total cotyledonary surface area [43].

### 2.5. Placental Microbiome Sample Collection

Sections of the chorioallantois were separated by blunt dissection to allow for tissue samples no greater than 5 cm^2^ of the ICM and allantois. Each sample was sectioned no more than 8 cm from the umbilical cord insertion site. Each tissue was briefly rinsed in sterile phosphate buffered saline (PBS) to remove debris due to natural delivery in the stock pen prior to trimming and dissection [44]. Cotyledons were selected from the middle of the gravid horn. Excess tissue was trimmed, and cotyledons were sectioned to fit within a 2 mL tube and then flash-frozen on dry ice. All samples were stored at −80 °C until further analysis.

### 2.6. Microbial DNA Extraction and Sequencing

Chemical and mechanical lysis was performed on all samples using the Precellys Evolution [45] (Bertin Instruments, Rockville, MD, USA). Microbial DNA was extracted from placental tissues (0.35 g) using the QIAmp PowerFecal kit (Qiagen, Germantown, MD, USA) following manufacturer protocols with the addition of the placement of PowerBead Pro tubes at 70 °C for 10 min following mechanical and chemical lysis.

Swab samples were prepared for microbial DNA extraction by sterilely cutting and placing whole swab heads in their respective bead tubes. Rumen fluid (0.25 g), along with vaginal and meconium swabs, were placed in bead tubes containing sterilized zirconia (0.3 g of 0.1 mm beads) and silicon (0.1 g of 0.5 mm beads) along with 1 mL of lysis buffer [45] (500 mM NaCl, 400 mM Tris-HCl, 50 mM EDTA, 4% SDS). Swab and rumen fluid samples underwent a DNA isolation step that included incubation at 70 °C for 10 min, repetition of the above lysis steps using 300 µL lysis buffer, and centrifugation to precipitate [46]. Microbial DNA was further purified within vaginal swab samples using the QIAamp PowerSoil Minikit (Qiagen), and the QIAamp Fast DNA Stool Mini Kit (Qiagen) was used for the rumen fluid and meconium. A Nanodrop ND-1000 spectrophotometer (NanoDrop Technologies, Wilmington, DE, USA) was used to determine DNA quality and quantity.

Microbial DNA from all samples was amplified using primers described in the literature [47] in order to amplify the hypervariable V4 region of the 16S rRNA gene (Supplementary File S1). Libraries were pooled at 30 ng/µL and sequenced via the MiSeq platform (Illumina). A positive control, where ZymoBIOMICS Microbial Community Standard (1 mM; Zymo Research, Irvine, CA, USA) substituted the DNA template in the reaction and a negative control where PCR-grade water substituted the DNA template were included in each step of library preparation.

### 2.7. Statistical Analysis

Statistical analyses of placental efficiency metrics, maternal BCS, maternal body weight, and calf body weight were performed in SAS v9.4 (SAS Institute Inc., Cary, NC, USA) using the PROC GLM procedure. Fixed effects included feeding treatment, mineral supplementation, and their interaction. Post-test pairwise comparisons were conducted using the LSD method of the LSMEANS procedure, where alpha was set at 0.05. Tendencies were considered when 0.05 < *p* ≤ 0.10.

### 2.8. Bioinformatics Analysis

Sequencing analysis was performed in QIIME2 v. 2020.8. [48], where quality filtering, denoising, and pairing were accomplished with the DADA2 plugin [49] in the University of Wyoming Advanced Computing Center Teton computing environment [50]. Alpha diversity indices, including Shannon’s index and Faith’s phylodiversity, were generated in QIIME2, and pairwise comparisons were conducted under Kruskal–Wallis permutational multivariate analysis of variance (PERMANOVA). These alpha diversity measurements account for the abundance and evenness of microbial taxa through Shannon’s index, while Faith’s phylodiversity accounts for phylogenetic differences within each community. Beta diversity indices, including weighted and unweighted UniFrac, were determined through PERMANOVA and visualized through Principal Coordinate Analysis (PCoA). The UniFrac distance metric accounts for the fraction of phylogenetic branch length that is shared between two collections of sequences [51]. As such, unweighted UniFrac more readily accounts for the relatedness and diversity of less dominant microbial taxa, while weighted UniFrac includes a mathematical weight that favors the more dominant microbial taxa within two given communities.

In order to maintain the maximum number of features and samples, three different comparisons were made: (1) a comparison of the placental membranes to each other; (2) a comparison of the vaginal microbiome over each gestational timepoint; and (3) comparisons of the placental and vaginal microbiomes with that of the fetal gut.

Taxonomic classification of the amplicon sequence variants (ASV) was performed within QIIME2 using the pretrained 16S classifier 515F/806R for the Silva 132 database [52]. Multiple comparisons were corrected for a false discovery rate (FDR). Under the Benjamini–Hochberg method, corrected *p*-values (*q*-values) less than 0.05 were considered significant. Tendencies were considered when 0.05 < *q* ≤ 0.10.

## 3. Results and Discussion

### 3.1. Placental Efficiency under Feed Intake Restriction

The number of cotyledons per placenta varied between 43 and 150. This was comparable to Bertolini et al., who observed a range of 52 to 153 cotyledons per placenta in Angus and Angus-cross pregnancies [53]. Placental weight ranged from 5.0 to 10.9 kg, and the total cotyledonary surface area ranged from 0.11 m^2^ to 0.41 m^2^. The averages for maternal weight, BCS, and placental efficiency characteristics for the main effects are listed in Table 1, and their averages for the interaction groups are listed in Table 2.

Consistent with previous findings [54], CON placentae tended to be more efficient than FR placentae (*p* = 0.10). Although only a tendency, a greater sample size or restricting the inclusion of study animals to within same age may have resulted in a true statistical difference. Because study animals were group fed, restriction was validated through BCS rather than individual intake. This is a limitation of the study, as individual intake restriction could not be confirmed. Recently, mineral supplementation has been implicated to aid placental adaptations in beef heifers when offered pre- and post-breeding [55]. Our data do not reflect these findings when mineral supplementation begins in late gestation, as no differences in placental efficiency were observed between S+ or S− pregnancies (*p* = 0.31). Although mineral supplementation has been suggested to improve gene expression regarding nutrient transport within the placenta during both low gestational weight gain pregnancies, only the CON/S+ diet displayed an increased placental efficiency compared to CON/S−, and no differences were observed between FR/S+ and FR/S− (*p* = 0.02).

Bovine placentomes are capable of growth in late gestation [54], suggesting that cattle may be able to compensate for FR during gestation by expanding the size of the cotyledonary surface [40]. The differences observed in placental efficiency within the present study are not easily explained by the number of small cotyledons, which did not differ between treatments (*p* = 0.39). The CON/S− group did display a lower percentage of small cotyledons compared to other interaction groups (*p* = 0.02), but CON/S+ remained similar to both FR/S+ and FR/S-, and no differences were detected between feeding treatments (*p* = 0.27). This may indicate that the compensatory growth of bovine placentomes is limited in late gestation. Although placental weights were not used to calculate placental efficiency, the fact that they did not differ between treatments (*p* = 0.36) may indicate that, at least in late gestation, the demand for nutrients within the placental organ is great enough for cotyledonary growth not to be a feasible compensatory mechanism for maternal FR.

### 3.2. The Placental Microbiome during Feed Restriction

#### 3.2.1. Main Effect of Feeding

No differences were detected in alpha diversity within the placental tissues of different feeding treatments (*q* ≥ 0.22). All three placental tissues of the CON calves differed from each other in weighted UniFrac (*q* ≤ 0.04), where the CON cotyledon was the most dissimilar (*q* = 0.02) and the CON allantois the least (*q* = 0.04). The greater degree of uniqueness within the cotyledon lends credence to the role it may play in transporting microbes to the placenta.

A greater degree of beta diversity similarity was observed in the FR placentae. The FR cotyledon failed to differ in unweighted UniFrac from the allantois (*q* = 0.11), and in weighted UniFrac, the FR cotyledon failed to differ from the ICM (*q* = 0.12). The similar beta diversity within the FR cotyledon may be in part due to compromised placental efficiency and metabolism. It has been reported that FR is often responsible for decreased placental efficiency [54], but a tendency toward a lower placental efficiency was noted in the present study. Our study was limited in the number of animals included in each treatment, which may account for the lack of significance in this metric. However, these findings indicate that a more efficient placenta may be responsible for a more robust and stable placental microbiome.

The FR allantois maintained the least unique microbial community within FR placentae. Because the allantois is the site of fetal waste deposition until the last ~20 days of gestation [56], this lack of microbial diversity may in fact be reflective of the fetal gut microbiome, which may contribute microbes to the allantois throughout gestation. Overall, the lack of microbial diversity observed in FR placentae implicate a less robust microbiome, which may interact in unknown ways with metabolic markers and signaling pathways affecting fetal health [55].

**Figure 1:**
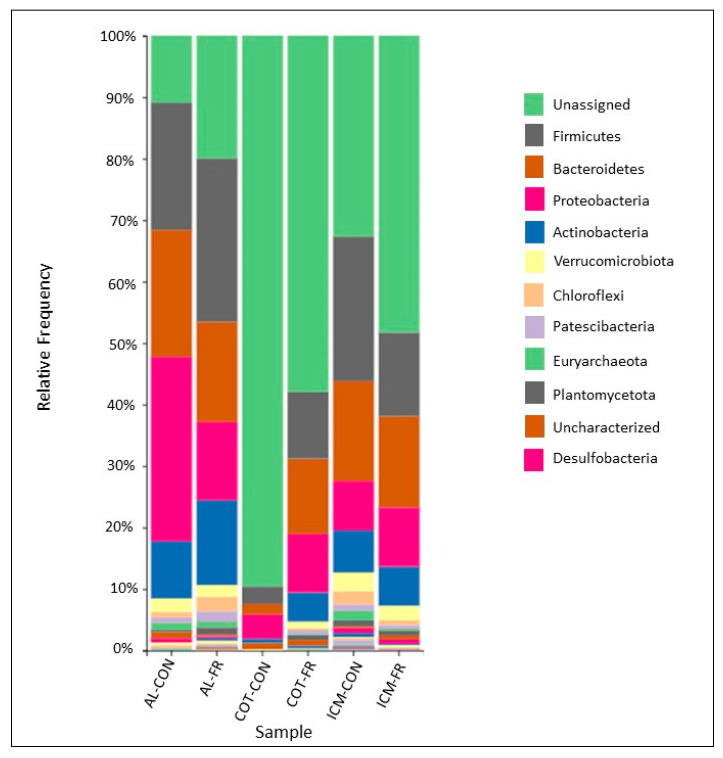
Qualitative phyla-level taxonomy plot of the placental microbiome by feeding treatment. The most relatively abundant taxa across all treatments and tissue types were the unassigned phyla, *Firmicutes*, *Bacteroidetes*, *Proteobacteria*, and *Actinobacteria*. Tissues include AL = allantois; ICM = intercotyledonary membrane of the chorion; COT = cotyledon. Treatments include CON = control diet; FR = 70% feed intake restricted diet.

Qualitative phyla-level taxonomic analysis (Figure 1) revealed that the relative abundances of specific phyla varied marginally by treatment, where *Firmicutes*, *Bacteroidetes*, *Proteobacteria*, and *Actinobacteria* dominated across the placental tissue types. Aside from *Actinobacteria*, these phyla do not differ from those reported in humans [57].

Historically, placental microbes have been regarded as the byproduct of contamination due to expulsion through the vaginal canal or an error in sample handling, exposing this tissue to contaminates through reagents or dissection tools [57,58]. In order to address this controversy, each placental sample was washed in PBS upon dissection with sterilized instruments [44], which ensured that the sampled microbes did not predominantly reside on the external surface of the placental membranes but were instead isolated from where they reside within the tissue itself [59]. As such, the placental microbes that were sampled were not exposed to the vaginal microbiome in the same way as the fetal oral cavity and gut during parturition. This is evidenced by the differing relative abundances of dominant phyla between the various placental tissues and the vagina. While surgical retrieval of the placenta would have combated these contamination concerns, we were unable to perform this procedure given the parameters of the current study.

The placental microbiome of humans has been noted to share a greater similarity with the maternal oral microbiome rather than that of vagina or feces [32]. Our study did not include an analysis of the oral microbiome, as ruminant species regurgitate ruminal contents and further chew feed, leading to similarities between these two microbial sites [20,60]. Therefore, comparisons with the oral microbiome were not included in the present study.

#### 3.2.2. Main Effect of Mineral Supplementation

No differences were detected in the alpha diversity within the placental tissues of different mineral treatments (*q* ≥ 0.34). Similar to feeding treatment, S+ and S− placentae showed little variation in the richness, abundance, and phylodiversity of microbes. Only the S+ cotyledon was more dissimilar than the allantois in both more (*q* = 0.05) and less (*q* = 0.02) dominant phyla. This illustrates a largely stable placental microbiome in late gestation that may be influenced by the maternal plane of nutrition, and to a lesser extent, mineral supplementation.

#### 3.2.3. Interactive Effect of Feeding and Supplementation

No differences were detected in the alpha diversity within the placental tissues of interaction groups (*q* ≥ 0.17). Although it was indicated that the CON/S+ treatment had the greatest placental efficiency (*p* = 0.02) and therefore would likely demonstrate a greater degree of microbial beta diversity, no differences were detected between the interaction groups in either of the UniFrac metrics (*q* ≥ 0.13). This further supports the claim that the plane of nutrition exerts a greater influence on the placental microbiome than mineral supplementation.

### 3.3. The Bovine Vaginal Microbiome during Feed Restriciton

#### 3.3.1. Main Effect of Feeding

The Alpha diversity of the vaginal microbiome at all three gestational timepoints indicated no differences in microbial richness (*q* ≥ 0.88) or phylodiversity (*q* ≥ 0.83). These data indicate that a stable, minimally variable vaginal microbiome exists in late gestation, corroborating previous findings [61,62]. This may account for the fact that FR had no impact on the pregnant bovine vaginal microbiome. No differences were detected between timepoints in either CON (*q* ≥ 0.51) or FR (*q* ≥ 0.17) for either UniFrac metric nor were any differences detected between the treatments at any timepoint (*q* ≥ 0.51).

#### 3.3.2. Main Effect of Mineral Supplementation

No differences were detected in alpha (*q* ≥ 0.34) or beta diversity (*q* ≥ 0.39) between gestational timepoints. The S+ and S− microbiomes failed to differ between timepoints in either of the UniFrac metrics (*q* ≥ 0.39). Individual mineral intake may not have been consistent, as mineral was provided ad libitum during group feeding. However, previous findings indicate that mineral supplementation during gestation fails to impact the vaginal microbiome [63], which this data confirm. Maternal mineral supplementation has been indicated to promote offspring health and performance [64], but it does not appear to do so by enhancing the vaginal microbiome.

#### 3.3.3. Interactive Effect of Feeding and Supplementation

**Figure 2:**
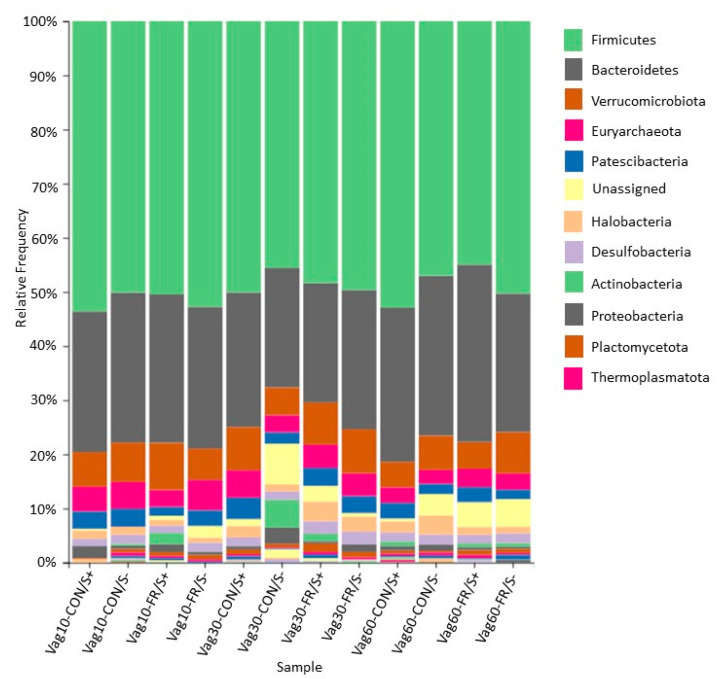
Qualitative phyla-level taxonomy plot of the microbiome of feed by mineral treatment groups for the vagina. The most relatively abundant taxa across all treatments and tissue types were *Firmicutes*, *Bacteroidetes*, and *Verrucomicrobiota*. Microbiome timepoints were taken at Vag60 = 60 days prior to parturition; Vag30 = 30 days prior to parturition; and Vag10 = 10 days prior to parturition. Treatments include CON/S+ = control diet with mineral supplementation; CON/S− = control diet without mineral supplementation; FR/S+ = 70% feed intake restricted diet with mineral supplementation; and FR/S− = 70% feed intake restricted diet without mineral supplementation.

No differences were detected in the alpha (*q* ≥ 0.39) or beta diversity (*q* ≥ 0.35) of any gestational timepoint between interaction groups. This lack of differences is reflected in the qualitative taxonomic analysis (Figure 2). *Firmicutes* (45–54%) and *Bacteroidetes* (22–33%) maintained the highest relative abundance in all treatments for each gestational timepoint. While the vaginal microbiome of women is dominated by *Lactobacillus* spp., (53), this is not the case in cattle [62], and the taxa observed in the present study agree with previous descriptions of the pregnant bovine uterus [65]. The high relative abundance of *Firmicutes*, and to a lesser degree *Bacteroidetes*, has been reported to prevent bacterial vaginosis in women [66], and this may be the role these microbes play in the ruminant microbiome. Where the vaginal microbiome of women maintains a low pH in order to prevent vaginosis [67], the composition of the ruminant vagina is near-neutral [65], but based on the phyla present within this microbial niche, may maintain a similar function.

Although women have demonstrated a diversity of core vaginal microbiomes, each represented by a distinct microbial composition [67], this does not seem to be the case in cattle [62], even within varying nutritional planes. Our data corroborate previous findings that indicate that the ruminant vagina displays a greater degree of bacterial diversity and a greater number of phyla than what is observed in women [68], many of which are associated with digestive tract microbes. The vaginal microbiome is less dynamic during pregnancy in women [69], a finding that our data also support during late gestation in cattle.

### 3.4. Contributions of the Vaginal Microbiome to the Fetal Gut

#### 3.4.1. Main Effect of Feeding

The vaginal microbiome has been suggested to contribute to the microbial load of the placenta [36] and fetal gut [37,38]. This may occur via the ascension of vaginal microbes to the intrauterine cavity, where they may be accessible to the fetus [36], as well as contact between the fetus and vaginal flora during natural delivery [70]. The fetal gut microbiomes are more diverse than the vaginal microbiome in Faith’s phylodiversity for both CON and FR (*q* ≤ 0.02). Alpha-diversity richness indicates that CON meconium does not differ from Vag10 (*q* = 0.12), while CON rumen fluid tended to be richer than Vag10 (*q* = 0.07) and was richer than both Vag30 and Vag60 (*q* ≤ 0.04). The FR fetal gut was richer than the vaginal microbiome at all gestational timepoints (*q* ≤ 0.02). The greater degree of richness and diversity seen at the alpha level in all fetal gut microbiomes indicates that, while a portion of gut microbes may be acquired from the vagina, this ecological niche cannot be solely responsible for inoculating the fetal gut microbiome. Beta diversity revealed the meconium and rumen fluid microbiome differed in the weighted and unweighted UniFrac of the vaginal microbiome at all timepoints (*q* ≤ 0.03) for both CON and FR pregnancies. These data further support differences in function between these microbiomes.

Feeding had little effect on any contribution the vaginal microbiome may have produced on the fetal gut. The similarity in alpha diversity richness observed between CON meconium and Vag10 was not reflected in FR pregnancies, and it is therefore not likely a product of contamination [20,58]. This finding may be compounded by the high levels of PCR inhibitors in meconium [23] or may illustrate a natural response to the inflammatory pathways that play a fundamental role in the onset of labor [71], allowing a greater degree of microbial richness to be shared between the meconium and vagina in CON pregnancies. More research would be required to determine any impacts that FR may have between the vaginal microbiome and the fetal gut.

#### 3.4.2. Main Effect of Mineral Supplementation

Shannon’s index revealed that S+ meconium and rumen fluid microbiomes were richer than the vaginal microbiome at all timepoints (*q* ≤ 0.05), but these differences were not present without mineral supplementation (*q* ≥ 0.77) for the meconium. The S− rumen fluid was richer than Vag30 and Vag60 (*q* = 0.05). Because mineral supplementation had no impact on the vaginal microbiome, it can be inferred that supplementation improved microbial richness within the fetal gut microbiome through another microbial niche, likely the placenta. Therefore, the placental microbiome may be one mode of action impacting calf performance through gestational mineral supplementation. [64].

Mineral supplementation created a greater degree of dissimilarity for both the dominant (*q* ≤ 0.02) and nondominant (*q* = 0.02) phyla of the meconium microbiome compared to the vaginal microbiome at all timepoints. This may further substantiate the favorable impact of mineral supplementation on the fetal gut microbiome. Both the meconium and the rumen fluid of S—pregnancies had a greater degree of similarities between the microbial communities than the vaginal microbiome displayed at all timepoints (*q* ≤ 0.03). Although mineral supplementation had no effect on the vaginal or placental microbiomes, these data indicate that in utero supplementation may improve the richness of the fetal gut microbiome, which may later influence calf performance [63]. These interactions remain unexplored but may be established up to 60 days prepartum.

#### 3.4.3. Interactive Effect of Feeding and Supplementation

Mineral supplementation was implicated to improve phylodiversity of the meconium microbiome in relation to the vagina. Faith’s phylodiversity revealed that CON/S+, CON/S−, and FR/S+ meconium tended to be more diverse than Vag30 and Vag60 (*q* = 0.08), where FR/S− meconium was similar to both timepoints (*q* = 0.30). Furthermore, the CON/S+, CON/S−, and FR/S− meconium was similar in phylodiversity to Vag10 (*q* ≥ 0.13), but FR/S+ meconium tended to be richer (*q* = 0.09). While the inclusion of a greater number of animals in each interaction group are necessary to confirm the significance of these findings, mineral supplementation may play a role in improving the alpha phylodiversity of the fetal gut microbiome. No differences were detected in Shannon’s richness within any interaction group (*q* ≥ 0.17).

Mineral supplementation did not have the same effect on the rumen fluid microbiome. The FR/S− rumen fluid tended to be richer in Faith’s phylodiversity than in Vag10 and Vag30 (*q* = 0.09) but was similar to Vag60 (*q* = 0.86), and FR/S+ rumen fluid tended toward similarity with all vaginal timepoints (*q* = 0.08). The rumen fluid of CON/S+ was similar in phylodiversity to Vag10 (*q* = 0.30) and tended to be similar to the other timepoints (*q* = 0.08), whereas the rumen fluid of CON/S− was similar to all vaginal timepoints (*q* ≥ 0.29).

Dominant taxa of the meconium microbiome were more dissimilar than those of Vag10 and Vag30 for all interaction groups (*q* ≤ 0.05). The relationship between the meconium microbiome and Vag60 varied in weighted UniFrac. Both CON/S− and FR/S+ were similar between microbiomes (*q* ≥ 0.12), FR/S− meconium tended to be more dissimilar (*q* = 0.08), and CON/S+ meconium was more dissimilar between communities (*q* = 0.03) than Vag60. The Vag60 microbiome represents the farthest recorded timepoint from parturition, which may account for this variability. Only the meconium of CON/S− was more dissimilar in less dominant phyla than the vaginal microbiome at all timepoints (*q* ≤ 0.03). These data do not reveal a clear influence of plane of nutrition or mineral supplementation in improving the diversity and health of the fetal gut microbiome in relation to vaginal microbes.

### 3.5. Shared Microbiota of the Fetal Gut and Placenta

#### 3.5.1. Main Effect of Feeding

The placental microbiome is characterized by its low abundance and low diversity [57]. Even compared to the small degree of diversity within the fetal gut microbiome [20], the cotyledon and allantois were less rich than rumen fluid for both feeding treatments (*q* ≤ 0.05). The richness of the ICM was most impacted by FR, as the CON ICM did not differ from the fetal gut microbiomes (*q* ≥ 0.31), but the FR ICM was less rich than rumen fluid (*q* = 0.02) and tended to be less rich than meconium (*q* = 0.07). The cotyledon was less diverse than both fetal gut microbiomes in FR pregnancies (*q* ≤ 0.03), and the allantois was less diverse than the fetal gut microbiome regardless of treatment (*q* ≤ 0.04). The FR ICM was less diverse than both fetal gut microbiomes (*q* ≤ 0.02) where the CON ICM was similar to the rumen fluid (*q* = 0.24).

The fetal gut microbiomes were unique to both the cotyledon (*q* ≤ 0.02) and ICM (*q* ≤ 0.04) in weighted and unweighted UniFrac. The rumen fluid of both CON and FR were different from the allantois in both metrics within the feeding treatments (*q* ≤ 0.02). The less dominant phyla of each respective allantois were similar to the FR meconium (*q* = 0.27) and tended to be similar to the CON meconium (*q* = 0.09).

The relationship between the cotyledon and fetal gut microbiomes was not affected by FR, which may indicate that a stable relationship exists between these microbial communities. This may be in part due to the cotyledon’s responsibility of perfusing the placental organ with blood [43] and providing a route for hematogenous transfer [32,33]. It was previously noted that FR decreases the diversity of the placental microbiome, and this appears to be most true in the relationship between the ICM and the fetal gut. The ICM is the most maternally facing membrane of the placenta [43], offering little interaction with the fetal gut. This divergent physiology may be responsible for the degree of differences indicated in FR pregnancies.

#### 3.5.2. Main Effect of Mineral Supplementation

Mineral supplementation enhanced the richness of both fetal gut microbiomes compared to the placental tissues (*q* ≤ 0.02). Both the fetal gut microbiomes of S− were similar in richness to the cotyledon (*q* ≥ 0.13), and S− meconium was similar to the allantois (*q* = 0.75) and ICM (*q* = 0.42). This extended to Faith’s phylodiversity, as both of the S+ fetal gut microbiomes were more diverse than the placental tissues (*q* ≤ 0.02). The S− cotyledon had a similar level of phylodiversity as the rumen fluid (*q* = 0.15), the S− allantois tended to be less diverse than both of the fetal gut microbiomes (*q* = 0.08), and the S− ICM was similar to both fetal gut microbiomes (*q* ≥ 0.15).

The fetal gut microbiomes of both supplementation treatments differed from the cotyledon in the weighted and unweighted Unifrac (*q* ≤ 0.04). This was also seen in the allantois (*q* ≤ 0.05) and ICM (*q* ≤ 0.01) of both treatments, although the S− ICM was similar to the meconium in unweighted UniFrac (*q* = 0.25).

Mineral supplementation did improve the richness and diversity of the fetal gut microbiome when compared to the placental microbiomes, where the relationship between the ICM and fetal gut was the most affected. The increased richness and diversity of the S+ fetal gut may help explain the improved growth performance of calves whose dams were supplemented during gestation [64].

#### 3.5.3. Interactive Effect of Feeding and Supplementation

No differences were observed between the placental tissues and the fetal gut microbiomes in either richness or phylodiversity for any interaction group (*q* ≥ 0.11). Meconium was similar to the cotyledon and allantois of all of the interaction groups in the weighted and unweighted UniFrac (*q* ≥ 0.11), except for the ICM, where both of the FR/S+ fetal gut microbiomes were dissimilar in both UniFrac metrics (*q* ≤ 0.05). Rumen fluid differed from the cotyledon and allantois (*q* ≤ 0.05) for all interaction groups in the weighted and unweighted UniFrac. However, the rumen fluid either tended to be dissimilar from the ICM in both of the UniFrac metrics (CON/S+ and FR/S-, *q* = 0.06) or was similar to the ICM in both metrics (CON/S− and FR/S+, *q* ≥ 0.25).

These data do not reveal a clear influence of plane of nutrition or mineral supplementation in improving the diversity and richness of the fetal gut microbiome in relation to the placental microbiome.

## 4. Conclusions

Our findings indicate that adequate maternal nutrition improved both placental efficiency and microbiome diversity. Mineral supplementation does not have an effect on the placental microbiome, but when combined with adequate feed intake, it improved placental efficiency. It is therefore advantageous to implement feeding strategies that increase placental efficiency, which not only contributes to greater calf performance in later life, but also aids in providing a richer and more unique placental microbiome.

Compromised feed intake tended to decrease placental efficiency in late gestation, which in turn decreased the diversity of the placental microbiome. The relationship between the cotyledonary microbiome and the fetal gut was least affected by the maternal environment, which may be due to the cotyledon’s responsibility to supply nutrients and potential microbes to the fetal environment.

Although mineral supplementation did not have an effect on the reproductive microbiomes, it was implicated to improve the richness and diversity of the fetal gut microbiome, substantiating prior research that gestational supplementation improves calf performance. Further research into the effects of mineral supplementation upon the fetal gut is warranted in order to guide feeding strategies on cow-calf operations. The impacts that maternal nutrition have upon the reproductive microbiome, and in turn, the fetal gut microbiome, may carry lifelong implications for health and performance. These data indicate a programming potential exists within the placental and fetal gut microbiomes, even in late gestation.

## Figures and Tables

**Table 1:** Analysis of body weight, BCS, and placental efficiency characteristics in all treatments from the general linear model (GLM) for main effects of feeding treatment and mineral supplementation.

	Feeding				Supplement			
	CON ^1^	FR ^1^	SE ^2^	*p*-Value	S+ ^3^	S− ^3^	SE ^2^	*p*-Value
Maternal BCS ^4^	5.98	5.57	0.14	0.05	5.73	5.82	0.14	0.64
Maternal Body Weight (kg)	671.45	625.78	18.49	0.09	653.08	644.15	18.49	0.73
Placental Weight (kg)	8.00	7.52	0.43	0.37	7.32	8.20	0.43	0.16
Calf Birth Weight (kg)	35.28	34.42	1.21	0.61	35.48	34.24	1.21	0.47
Number Cotyledons	81.36	99.21	7.99	0.12	93.21	87.36	7.99	0.60
Number small ^5^ cotyledons	52.29	70.94	8.15	0.11	66.36	56.87	8.15	0.41
Percent small ^5^ cotyledons	62.53	68.43	3.74	0.27	68.79	62.17	3.74	0.21
Cotyledonary surface area (m^2^)	0.21	0.23	0.02	0.61	0.21	0.23	0.02	0.47
Placental efficiency (kg/m^2^)	178.83	154.07	10.43	0.10	173.96	158.93	10.43	0.31

^1^ CON = control diet; FR = 70% feed intake restricted diet; ^2^ SE = standard error; ^3^ S+ = mineral supplementation provided, S− = no mineral supplementation provided; ^4^ BCS = body condition score. ^5^ Small cotyledons defined as <30 cm^2^.

**Table 2:** Analysis of body weight, BCS, and placental efficiency characteristics in all treatments from the general linear model (GLM) for interactions between feeding treatment and mineral supplementation.

	Feeding × Mineral Supplement Interaction ^1^					
	CON/S+ ^1^	CON/S− ^1^	FR/S+ ^1^	FR/S− ^1^	SE ^2^	*p*-Value ^3^
Maternal BCS ^4^	6.11 ^a^	5.86 ^abx^	5.36 ^by^	5.79 ^ab^	0.21	0.09
Maternal Body Weight (kg)	677.67	665.23	628.48	623.08	27.14	0.89
Placental Weight (kg)	7.84	8.16	6.80	8.24	0.63	0.36
Calf Birth Weight (kg)	35.96	34.60	34.99	33.87	1.78	0.42
Number Cotyledons	84.00	78.71	102.43	96.00	11.72	0.96
Number small ^5^ cotyledons	62.00	42.57	70.71	71.17	11.96	0.39
Percent small ^5^ cotyledons	72.32 ^a^	52.73 ^b,y^	65.25 ^ab,x^	71.61 ^a^	5.48	0.02
Cotyledonary surface area (m^2^)	0.18 ^ax^	0.25 ^a^	0.24 ^ax^	0.21 ^ay^	0.03	0.04
Placental efficiency (kg/m^2^)	205.28 ^ax^	152.37 ^b^	142.65 ^b^	165.48 ^aby^	15.31	0.02

^1^ CON/S+ = control diet and mineral supplementation provided; CON/S− = control diet and no mineral supplementation provided; FR/S+ = 70% feed intake restricted diet and mineral supplementation provided; FR/S− = 70% feed intake restricted diet and no mineral supplementation provided; ^2^ SE = standard error; ^3^ *p*-value of interaction term between feed and mineral supplementation. Superscript indicates differences (a, b; *p* ≤ 0.05) or tendencies (x, y; 0.05 < *p* ≤ 0.10). ^4^ BCS = body condition score. ^5^ Small cotyledons defined as <30 cm^2^.

## Data Availability

The datasets generated during and/or analyzed during the current study are available from the corresponding author upon reasonable request.

## Supplementary Materials

The following are available online at https://www.mdpi.com/article/10.3390/ani11082219/s1, File S1: Additional Protocol Information.
