# Supplementary material for: Late Gestation Maternal Feed Restriction Decreases Microbial Diversity of the Placenta While Mineral Supplementation Improves Richness of the Fetal Gut Microbiome in Cattle
Source: Animals (Basel). 2021 Jul 27;11(8):2219. doi: 10.3390/ani11082219 (PMC8388467; doi:10.3390/ani11082219)
Supplement: Supplementary file 1 [file animals-11-02219-s001.zip › animals-1299374-supplementary.pdf]

Supplementary File

Microbial DNA Library Preparation PCR Reaction 1:

Each reaction contained:

- 2.5 µL of genomic DNA (5 ng/µL)
- 5 µL forward and reverse amplicon primer (1 µM each)
- 12.5 µL of 2 X KAPA HiFi HotStart Ready Mix (Kapa Biosystems, Inc., Wilmington, MA, USA)

PCR Protocol:

- All steps performed in Mastercycler thermal cycler (Eppendorf, Hamburg, Germany)
- A first amplification: denaturation at 95°C for 3 min, followed by 25 cycles of denaturing (95°C for 30 s), annealing (60°C for 30 s) and extension (72°C for 30 s), with a final extension at 72°C for 5 min.
- Amplicons and controls were then cleaned with Agencourt AMPure XP beads (Beckman, Coulter Brea, CA, USA).

Microbial DNA Library Preparation PCR Reaction 2:

Each reaction contained:

- 2 µL Nextera XT forward and reverse index primers (Illumina, San Diego, CA, USA)
- 5 µL amplicon DNA
- 25 µL of 2 X KAPA HiFi HotStart Ready Mix
- 16 µL PCR-grade water.

PCR Protocol

- All steps performed in Mastercycler thermal cycler (Eppendorf)
- Samples were denatured at 95°C for 3 min, followed by 8 cycles of a denaturing (95°C for 30 s), annealing (60°C for 30 s), and extension step (72°C for 30s), with a final extension at 72°C for 5 min.
- Libraries were pooled at 30 ng/µL and then sequenced via the MiSeq platform (Illumina).
